# Effect of perceived autonomy supports on exercise persistence for adolescents: an integrated model based on basic psychological needs theory and the theory of planned behavior

**DOI:** 10.3389/fpsyg.2025.1692940

**Published:** 2025-12-11

**Authors:** Xuening Li, Jianyong Zhu, Huasen Yu, Jiabin Lin

**Affiliations:** 1 Department of Physical Education, Nanjing University of Posts and Telecommunications, Nanjing, China; 2 College of Physical Education and Health, East China Normal University, Shanghai, China; 3 College of Physical Education, Changchun Normal University, Changchun, China

**Keywords:** perceived autonomy support, exercise persistence, adolescent, basic psychological needs, theory of planned behavior

## Abstract

**Introduction:**

Although previous research has confirmed a positive association between perceived autonomy support and adolescents’ exercise persistence, the psychological mechanisms underlying this relationship remain insufficiently explored. The study aims to investigate the relationship and mechanisms linking perceived autonomy support to exercise persistence in adolescents, based on the integrated framework of basic psychological needs (BPN) and the theory of planned behavior (TPB).

**Methods:**

Using a simplified stratified random sampling method, 4,345 adolescents aged 10–20 years completed measures of perceived autonomy support, BPN, TPB, and exercise persistence. Structural equation modeling (SEM) was constructed to test the effectiveness of the integrated model and examine hypothesized pathways.

**Results:**

SEM analysis showed that perceived autonomy support directly predicted exercise persistence. This relationship was also partially mediated via BPN alone and by the chained mediation of BPN and TPB constructs. Furthermore, this indirect effect was moderated by educational stage and gender. For junior high school students, behavioral attitudes did not significantly influence behavioral intentions. Both behavioral intentions and perceived behavioral control positively affected exercise persistence, whereas perceived behavioral control did not significantly influence exercise persistence among female senior high school students.

**Conclusion:**

These findings confirm the applicability of integrating BPN and TPB to explain how exercise support environment foster adolescents’ exercise persistence. They further highlight the importance of tailoring interventions strategies to differences in educational stage and gender. Further research should build on this integrated model to design stage- and gender- specific interventions and examine their longitudinal effectiveness across different developmental periods.

## Introduction

Adolescent health challenges are becoming increasingly urgent. According to the World Health Organization (2020), approximately 81% of adolescents globally experience a decline in physical health, with nearly one-fifth classified as overweight or obese. China’s eighth national survey (2019) similarly indicates that problems such as myopia and obesity among Chinese adolescents remain unresolved (China Youth Daily, 2022). Regular physical activity is widely recognized as one of the most effective approaches to improving physical and mental health (Glowacki et al., 2017), yielding benefits such as better emotional stability, enhanced stress regulation, and reduced chronic disease risk (Chow et al., 2022; Varanoske et al., 2022). Despite these advantages, insufficient physical activity remains a pervasive global public health concern (Guthold et al., 2018). The World Health Organization’s (2019) Global Physical Activity Report revealed that 80% of 1.6 million adolescents failed to meet the recommended daily exercise levels. Therefore, identifying the determinants of adolescents’ exercise persistence is a critical research priority.

Among these determinants, perceived autonomy support—defined as perception of contextual factors that encourage self-directed activity—has emerged as a significant predictor of sustained exercise engagement (Moustaka et al., 2012; Fang et al., 2020). Although existing studies have demonstrated a positive association between perceived autonomy support and exercise persistence (Behzadnia et al., 2018; Moreno-Murcia et al., 2022), the underlying psychological mechanisms driving this relationship remain insufficiently explored.

Extant research suggests that both basic psychological needs (BPN) theory and the theory of planned behavior (TPB) each offer explanatory value for adolescents’ exercise persistence. TPB, which emphasizes belief-based perceptions, focuses on the formation of behavioral intentions and has demonstrated strong predictive validity in this domain (Yang et al., 2016). In contrast, BPN theory highlights the satisfaction degree of need satisfaction that shapes individuals’ expectations and behavioral choices (Zhang and Chen, 2001). However, BPN theory does not adequately explain how the satisfaction of basic needs translates into behavioral intentions and actions, whereas BPN theory pays limited attention to the motivational foundations of these intentions (Yin et al., 2018). Integrating the two theories may thus provide a more complete social–cognitive account of how contextual support shapes exercise persistence than either model alone (Gucciardi and Jackson, 2015). Despite growing interest in integrating BPN and TPB, no study has yet investigated whether these constructs function as a sequential mediating pathway between perceived autonomy support and exercise persistence, or whether this pathway differs by educational stages or gender. Addressing these gaps is essential for developing tailored interventions that effectively enhance exercise persistence in diverse adolescent populations.

BPN, a core concept of self-determination theory (SDT), is widely recognized as essential to individuals’ well-being and the maintenance of sustained behavior (Deci and Ryan, 2017). BPN encompasses three dimensions, autonomy (the will and willingness to engage in an activity), competence (the perceived capability to successfully perform the activity), and relatedness (the experience of care, belonging, and social significance through connections with others), all of which are critical for psychosocial functioning (Ryan and Deci, 2000). According to SDT, contextual factors determine the degree to which these needs are satisfied, with autonomy-supportive environments playing a pivotal role (Deci and Ryan, 2000). Empirical evidence indicates that when students perceive strong autonomy support from physical education teachers, their basic needs are more fully met, leading to more positive attitudes and greater persistence in exercise (Standage et al., 2012). Likewise, support from family and peers enhances high school athletes’ BPN satisfaction, with higher levels of need fulfillment associated with perceived support from multiple significant others (Amorose et al., 2016). Satisfying exercise-related psychological needs is also a key determinant of adolescents’ exercise persistence (Zhang and Dong, 2017), as the internal motivational forces driving perseverance depend on the fulfillment of these needs (Wilson et al., 2006). Consistent with this view, BPN satisfaction has been shown to strengthen adolescents’ long-term exercise intentions (Chen et al., 2006), and recent longitudinal research confirms that higher BPN satisfaction predicts greater exercise persistence over a six-month period (Kang et al., 2020). In summary, evidence indicates that BPN plays a role in how perceived autonomy support contributes to exercise persistence.

TPB is widely regarded as one of the most influential models for explaining intentional behavior (Abraham and Sheeran, 2003). According to TPB, behavioral performance is determined by behavioral intention, which in turn is shaped by three antecedents: attitude, perceived behavioral control, and subjective norm. In sport and exercise contexts, TPB has been extensively applied to predict exercise behavior, exercise intention, and overall physical activity levels (Gomes et al., 2018; Kang and Wang, 2016), with consistent evidence showing that each antecedent significantly contributes to the formation of intention and subsequent behavioral enactment. Strengthening exercise intention is vital for promoting long-term engagement in physical activity (Jouper and Hassmén, 2009). For instance, a longitudinal study of 437 gym users found that behavioral intention significantly predicted later exercise persistence (Rodrigues et al., 2020). Moreover, when individuals perceive constrains related to resources or abilities required for a behavior, perceived behavioral control may exert a direct effect on actual behavior (Ajzen and Schmidt, 2020). During the early stages of exercise engagement, fostering perceived behavioral control may even be more influential than intention itself (Anderson and Lavallee, 2008). Consistent with this, both behavioral intention and perceived behavioral control have been identified as key predictors of exercise persistence (Husebø et al., 2013). However, the predictive power of TPB may vary across demographic moderator such as gender and educational stages (Ajzen, 1991; Wang and Zheng, 2022), and existing findings on these moderating effects remain inconclusive (Nie and Dong, 2015; Viksi and Tilga, 2022). Taken together, these lines of research offer a deeper understanding of TPB and provide valuable insights into factors that shape individual’s exercise persistence.

Furthermore, satisfying BPN plays a pivotal role in enhancing individuals’ behavioral intention by fostering positive attitudes toward the behaviors, aligning actions with perceived social norms, and enhancing perceived behavioral control (Lei et al., 2022). Studies have demonstrated that BPN indirectly affects exercise behavior through the mediating pathways of TPB (Cho et al., 2023; Harris and Hagger, 2007). A longitudinal investigation revealed that higher levels of BPN satisfaction were associated with more positive attitudes, stronger perceived behavioral control, and more favorable subjective norms, which in turn predicted stronger behavioral intentions; both intentions and perceived behavioral control subsequently contributed to exercise persistence (Gucciardi and Jackson, 2015). This aligns with the cognitive decision-making model of exercise persistence, which suggests that satisfying BPN clarifies and consolidates intentions to exercise, thereby increasing the likelihood of sustained long-term engagement (Chen et al., 2006). In summary, evidence indicates that BPN is directly and positively associated with TPB antecedents.

As noted above, although existing evidence examining the roles of BPN and TPB in the link between perceived autonomy support and exercise persistence, these variables have rarely been investigated as components of an integrated, interactive system. Moreover, little research has explored whether the mediating pathways differ across educational stages or gender. Thus, this study aims to investigate the psychological mechanism of the effect of perceived autonomy support and exercise persistence among adolescents. To this end, we propose the following hypotheses:

*H1: Perceived autonomy support positively predicts exercise persistence and BPN;*

*H2: BPN positively predict TPB antecedents and exercise persistence;*

*H3: TPB antecedents positively predict behavioral intention;*

*H4: Perceived behavioral control and behavioral intention positively predict exercise persistence;*

*H5: Perceived autonomy support has an indirect effect on TPB antecedents through fostering the satisfaction of BPN;*

*H6: BPN have an indirect effect on behavioral intention through TPB antecedents;*

*H7: TPB antecedents have an indirect effect on exercise persistence through behavioral intention;*

*H8: BPN have an indirect effect on exercise persistence through perceived behavioral control;*

*H9: Perceived autonomy support has an indirect effect on exercise persistence through fostering the satisfaction of BPN;*

*H10: Perceived autonomy support improves exercise persistence through serial indirect effects of BPN and TPB;*

*H11: The indirect pathways differ across educational levels;*

*H12: The indirect pathways differ between male and female adolescents.*

**Figure 1:**
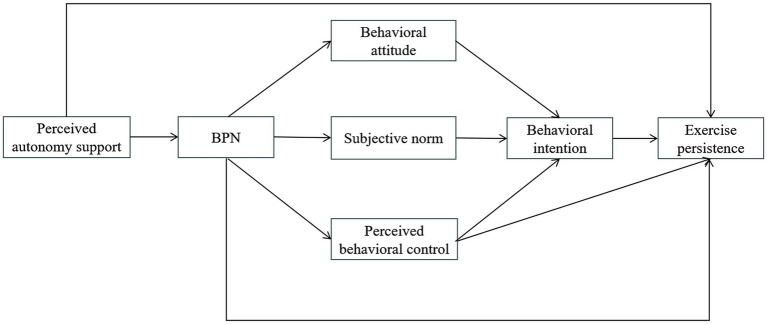
Hypothetical model.

Hypothetical model depicted in Figure 1 to reveal the underlying mechanism.

## Methods

### Participant and procedure

According to World Health Organization (2020), adolescence spans ages 10–19. This study focused on the students within this age range enrolled in junior and senior high schools. From June 1 to September 18, 2024, we employed a simplified class-level cluster-sampling strategy to administer questionnaires across four Chinese provinces (Hebei, Henan, Jilin, and Liaoning). In each region, two junior high schools (grades 7–9) and two senior high schools (grades 10–11) were randomly selected; grade-12 students were excluded due to examination pressures. Within each selected school, three classes per grade were surveyed, resulting in 4,776 returned questionnaires. After excluding 431 invalid responses characterized by regular answering patterns or faulty data, 4,345 valid responses remained, yielding a valid response rate of 90.98%. The final sample comprised 854 seventh graders (417 males, 437 females), 837 eighth graders (410 males, 427 females), 866 ninth graders (430 males, 436 females), 874 tenth graders (424 males, 450 females), and 914 eleventh graders (453 males, 461 females), with balanced gender distributions across grades. Participants ranged in age from 10 to 20 years (*M* = 15.30; *SD* = 1.3).

This study was approved by the University Committee on Human Research Protection (HR 284–2024). Prior to participation, all participants were fully informed of the study’s purpose as well as the confidentiality and anonymity of their responses. Written informed consent was obtained from both participants and their legal guardians. All procedures adhered strictly to the revised ethical principles of the Declaration of Helsinki.

### Measure

BPN satisfaction was measured using the 21-item Basic Psychological Need in Exercise Scale (BPNES) originally developed by Vlachopoulos et al. (2011). The present study employed the Chinese version validated by Liu et al. (2013). The scale consists of three dimensions, competence, autonomy, and relatedness, and adopts a Likert 7-point scale (from 1 = “strongly disagree” to 7 = “strongly agree”), with higher scores indicating higher satisfaction with basic needs. This scale has good internal consistency, with Cronbach’s *α* = 0.920, for the overall scale and *α* = 0.913, 0.881, and 0.875 for competence, autonomy, and relatedness, respectively. Confirmatory factor analysis (CFA) supported the intended three-factor structure: χ^2^/df = 3.459, GFI = 0.982, NFI = 0.986, IFI = 0.989, CFI = 0.989, and RMSEA = 0.050, all indices indicating good model fit.

Perceived autonomy support was assessed using the scale developed by Fang et al. (2020), which comprises three dimensions: information atmosphere (3 items), institutional facilities (4 items), and physical environment (3 items). Items are rated on a 5-point Likert scale (1 = “completely disagree” to 5 = “completely agree”), with higher scores indicating stronger perceived support for autonomous exercise behavior. The scale is publicly accessible. In this study, the overall Cronbach’s *α* was 0.926, and subscale reliabilities ranged from 0.847 to 0.904. CFA results confirmed good model fit: χ^2^/df = 3.709, GFI = 0.990, NFI = 0.993, IFI = 0.994, CFI = 0.994, and RMSEA = 0.051.

Exercise persistence was evaluated using the 14-item questionnaire developed by Wang et al. (2016), which assesses behavioral habits, effort commitment, and emotional experience. Each item is scored on a Likert 5-point scale (1 = “totally disagree” to 5 = “totally agree”), with higher scores reflecting greater exercise persistence. The scale is freely accessible. Internal consistency in the present study was excellent (overall *α* = 0.941; subscales α = 0.830–0.911). CFA supported the three-dimensional structure with excellent fit indices: χ^2^/df = 4.093, GFI = 0.958, NFI = 0.981, IFI = 0.985, CFI = 0.985, and RMSEA = 0.048.

Exercise-related cognitions were assessed using the TPB questionnaire developed by Hagger et al. (2002), The Chinese version, which has demonstrated good validity (Anderson and Lavallee, 2008), is also publicly available. The scale includes 10 items covering four dimensions: behavioral intention, behavioral attitude, perceived behavioral control, and subjective norm, rated on a 7-point Likert scale. In this study, the Cronbach’s α for overall scale was 0.902, and for each of the three subscales, it ranged from 0.799 to 0.937, respectively. CFA showed good model fit: χ^2^/df = 4.001, GFI = 0.979, NFI = 0.986, IFI = 0.988, CFI = 0.988 and RMSEA = 0.047.

### Statistical analysis

The full path model which included BPN, exercise persistence, TPB, and self-efficacy, was used for power analysis. With four degrees of freedom, a minimum sample size of 829 participants was required to achieve a close fit (RMSEA = 0.06) at 80% power (Kim et al., 2015). The current study far exceeded this requirement, with 4,345 participants, ensuring sufficient statistical power.

Following questionnaires collection, data were analyzed using a two-step process. First, preliminary analyses were performed using SPSS 21.0. These included internal consistency testing for all scales and a common method deviation test using Harman’s single-factor method. Descriptive statistics (means, standard deviations, Cronbach’s *α*) were computed; Pearson correlations were then used to explore associations among perceived autonomy support, BPN, behavioral intention, perceived behavioral control, behavioral attitude, subjective norm, and exercise persistence. Independent-sample t-tests were performed to assess gender differences in each variable. Second, structural equation modeling (SEM) was employed to test the hypothesized serial multiple-mediation model, evaluating how perceived autonomy support influences exercise persistence. Model fit was assessed using goodness-of-fit indices. The chi-square to degrees of freedom ratio (χ^2^/df) was evaluated, with values between 2 and 5 considered acceptable (Bentler, 1990). The goodness-of-fit index (GFI), normed fit index (NFI), comparative fit index (CFI), and incremental fit index (IFI) were also considered, with values of 0.90 or higher denoting acceptable fit (Ramírez et al., 2025). Finally, the Root Mean Square Error of Approximation (RMSEA) was used to evaluate model fit per degree of freedom (Byrne, 2013), where values less than 0.05 indicate a close fit and those between 0.05 and 0.08 indicate an acceptable model fit (Sharma et al., 2005). All SEM analysis were conducted in AMOS 26.0 using maximum-likelihood estimation. Indirect effects were tested using 5,000 bias-corrected bootstrap resamples to generate 95% confidence interval (CI). Statistical significance was set at *p* < 0.05.

## Results

### Common method bias test

Given that all data were self-reported, common method bias was assessed using Harman’s single-factor test (Podsakoff et al., 2003). An unrotated principal-component analysis of all items extracted ten factors with eigenvalues greater than one, with the first factor accounting for just 20.87% of the total variance. This result indicates that common method bias was not a substantial threat in the current study (Table 1).

**Table 1:** Demographic differences in perceived autonomy support, BPN, behavioral intention, perceived behavioral control, behavioral attitude, subjective norm, and exercise persistence (*N* = 4,345).

Variable	Perceived autonomy support	BPN	Behavioral intention	Perceived behavioral control	Behavioral attitude	Subjective norm	Exercise persistence
	M ± SD	M ± SD	M ± SD	M ± SD	M ± SD	M ± SD	M ± SD
Gender							
Male	35.63 ± 9.23	64.02 ± 13.59	14.84 ± 4.54	14.51 ± 13.13	14.43 ± 5.24	15.30 ± 4.53	52.29 ± 12.10
Female	33.85 ± 8.85	60.35 ± 14.01	12.76 ± 4.51	13.13 ± 4.65	12.97 ± 5.24	14.68 ± 4.40	48.58 ± 12.06
t	6.51***	8.77***	7.89***	9.77***	9.19***	1.64***	10.13***
Educational stage							
Junior	35.93 ± 9.16	65.13 ± 13.08	14.23 ± 4.09	14.40 ± 4.56	14.29 ± 5.25	15.73 ± 4.35	52.85 ± 11.72
Senior	33.00 ± 8.68	57.90 ± 13.99	11.95 ± 4.85	12.95 ± 4.74	12.83 ± 5.22	13.91 ± 4.43	46.89 ± 12.07
t	10.59***	17.42***	16.70***	10.18***	8.99***	13.52***	10.59***

*p* < 0.05; **p < 01; *** *p* < 0.001.

### Demographic differences among variables

As shown in Table 1, t-test results indicated gender differences. Male students reported higher levels of perceived autonomy support (*t* = 6.51, *p* < 0.001), BPN satisfaction (*t* = 8.77, *p* < 0.001), behavioral intention (*t* = 7.89, *p* < 0.001), perceived behavioral control (*t* = 9.77, *p* < 0.001), behavioral attitude (*t* = 9.19, *p* < 0.001), subjective norm (*t* = 1.64, *p* < 0.001), and exercise persistence (*t* = 10.13, *p* < 0.001) compared with female students. Regarding educational stage, junior high school students scored significantly higher than senior high school students on perceived autonomy support (*t* = 10.59, *p* < 0.001), BPN satisfaction (*t* = 17.42, *p* < 0.001), behavioral intention (*t* = 16.70, *p* < 0.001), perceived behavioral control (*t* = 10.18, *p* < 0.001), behavioral attitude (*t* = 8.99, *p* < 0.001), subjective norm (*t* = 13.52, *p* < 0.001), and exercise persistence (*t* = 10.59, *p* < 0.001).

### Descriptive statistics and Pearson correlation coefficient among variables

Table 2 presents the correlation among all study variables. All variables were positively correlated, with coefficients ranging from 0.39 to 0.73. These results provide a solid theoretical basis for examining the mediating role of BPN, behavioral intention, perceived behavioral control, behavioral attitude, subjective norm in the relationship between perceived autonomy support and exercise persistence.

**Table 2:** Correlation analysis of perceived autonomy support, BPN, behavioral intention, perceived behavioral control, behavioral attitude, subjective norm, and exercise persistence in adolescents (*N* = 4,345).

Variable	M ± SD	1	2	3	4	5	6	7
1. BPN	62.15 ± 13.92	1	–	–	–	–	–	–
2. Behavioral intention	13.29 ± 4.55	0.47**	1	–	–	–	–	–
3. Behavioral attitude	13.69 ± 5.29	0.43**	0.45**	1	–	–	–	–
4. Subjective norm	14.98 ± 4.47	0.49**	0.42**	0.44**	1	–	–	–
5. Perceived behavioral control	13.81 ± 4.69	0.52**	0.50**	0.65**	0.48**	1	–	–
6. Exercise persistence	50.40 ± 12.22	0.72**	0.53**	0.53**	0.52**	0.60**	1	–
7. Perceived autonomy support	34.73 ± 9.08	0.62**	0.40**	0.39**	0.43**	0.48**	0.73**	1

*p* < 0.05; **p < 01; *** *p* < 0.001.

### Stepwise regression analysis

Stepwise regression analyses were conducted to examine the mediating roles of BPN, behavioral intention, perceived behavioral control, behavioral attitude and subjective norm in the relationship between perceived autonomy support and exercise persistence for junior and senior high school students. As shown in Tables 3, 4, the full set of predictors accounted for 69.60% of the variance in exercise persistence for junior high school students and 68.10% for senior high school students (see Tables 3, 4). For senior high school students, once behavioral intention and the remaining variables were entered into the model, subjective norms no longer showed a statistically significant effect, whereas gender emerged as a significant predictor. This pattern suggests that the influence of subjective norms on exercise persistence may be moderated by gender or that other predictors exert a more direct effect on exercise persistence, thereby reducing the explanatory contribution of subjective norms.

**Table 3:** Stepwise regression analysis of study variables for junior high school students.

Variables	Step 1			Step 2			Step 3		
	B	SE	β	B	SE	β	B	SE	β
Control variables									
Age	0.46	0.27	0.05	0.07	0.18	0.01	−0.15	0.16	−0.02
Gender	−2.34	0.46	−0.10***	−1.07	0.30	−0.05***	−0.64	0.27	−0.03**
Grade level	−0.23	0.41	−0.02	−0.10	0.26	−0.01	0.04	0.24	0.01
Independent variables									
Perceived autonomy support				0.97	0.02	0.76***	0.68	0.02	0.53***
BPN							0.32	0.01	0.36***
*R* ^2^	0.01			0.58			0.66		
Variables	Step 5			Step 5			Step 6		
	B	SE	β	B	SE	β	B	SE	β
Control variables									
Age	−0.09	0.15	−0.01	−0.01	0.15	−0.001	−0.01	0.15	0.01
Gender	−0.38	0.26	−0.02	−0.35	0.26	−0.015	−0.31	0.26	−0.01
Grade level	0.01	0.23	0.01	−0.05	0.23	−0.01	−0.01	0.23	−0.01
Independent variables									
Perceived autonomy support	0.60	0.02	0.47***	0.59	0.02	0.46***	0.58	0.02	0.45***
BPN	0.27	0.01	0.30***	0.24	0.01	0.27***	0.23	0.01	0.26***
Perceived behavioral control	0.49	0.04	0.19***	0.29	0.04	0.11***	0.24	0.04	0.09***
Behavioral attitude				0.17	0.03	0.08***	0.15	0.03	0.07***
Subjective norms				0.28	0.04	0.10***	0.26	0.04	0.10***
Behavioral intention							0.22	0.04	0.08***
*R* ^2^	0.68			0.69			0.70		

*p* < 0.05; **p < 01; *** *p* < 0.001.

**Table 4:** Stepwise regression analysis of study variables among senior students.

Variables	Step 1			Step 2			Step 3		
	B	SE	β	B	SE	β	B	SE	β
Control variables									
Age	−1.53	0.23	−0.15***	−0.48	0.18	−0.05***	−0.24	0.16	−0.02
Gender	−5.50	0.50	−0.23***	−3.47	0.43	−0.15***	−2.24	0.37	−0.1***
Grade level	−0.75	0.55	−0.02	−0.18	0.42	−0.01	−0.48	0.36	0.02
Independent variables									
Perceived autonomy support				0.88	0.03	0.63***	0.54	0.03	0.39***
BPN							0.40	0.02	0.46***
*R* ^2^	0.08			0.46			0.60		
Variables	Step 5			Step 5			Step 6		
	B	SE	β	B	SE	β	B	SE	β
Control variables									
Age	−0.10	0.15	−0.01	−0.05	0.14	−0.01	0.02	0.14	0.01
Gender	−1.16	0.26	−0.07***	−1.32	0.34	−0.06***	−1.18	0.33	−0.05***
Grade level	0.35	0.23	0.02	−0.35	0.33	−0.02	−0.47	0.32	−0.02
Independent variables									
Perceived autonomy support	0.47	0.02	0.34***	0.44	0.02	0.31***	0.43	0.02	0.31***
BPN	0.32	0.02	0.38***	0.30	0.02	0.34***	0.28	0.02	0.32***
Perceived behavioral control	0.61	0.04	0.24***	0.35	0.05	0.14^***^	0.30	0.05	0.12***
Behavioral attitude				0.47	0.04	0.20^***^	0.41	0.04	0.17***
Subjective norms				0.11	0.04	0.04^*^	0.05	0.04	0.02
Behavioral intention							0.33	0.04	0.13***
*R* ^2^	0.64			0.67			0.68		

*p* < 0.05; **p < 01; *** *p* < 0.001.

### Mediation analysis

We constructed separate SEM models for junior and senior high school students using AMOS 26.0. Since exercise persistence among junior high school students was not influenced by control variables, these students were analyzed as a single cohort. In contrast, senior high school students showed significant gender differences in exercise persistence; therefore, three models were tested for this group: the overall sample, males only, and females only. A shown in Table 5, all models met the recommended fit-index criteria, indicating good model fit. Mediation effects were tested using the bias-corrected percentile bootstrap method with 5,000 resamples, and 95% confidence intervals (CI) were calculated. A mediation effect was deemed significant when its 95% CI did not include zero.

**Table 5:** The fitting results of the structural equation model.

Fit-index	Junior	Senior		
		Overall sample	Male	Female
χ^2^/df	4.610	5.424	4.542	4.918
RMSEA	0.070	0.050	0.064	0.066
GFI	0.912	0.952	0.921	0.917
CFI	0.950	0.973	0.959	0.946
IFI	0.950	0.973	0.959	0.946
NFI	0.947	0.968	0.949	0.933
TLI	0.941	0.967	0.951	0.935

#### Mediation analysis for the overall junior and senior high school students

**Figure 2:**
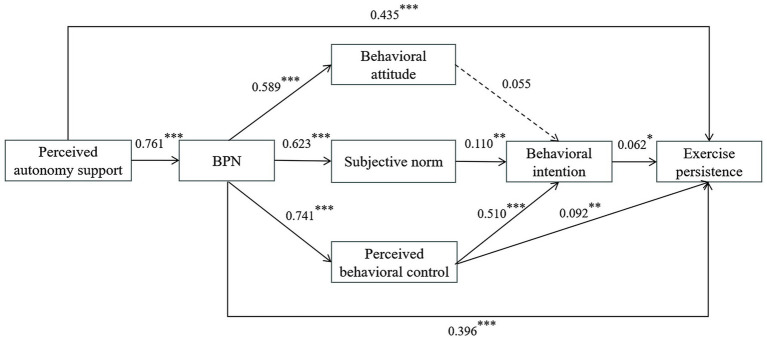
Standardized path coefficients of the SEM of hypothesized relationships between variables in the model for junior high school students.

As shown in Figure 2, the SEM results for junior high school students indicated that all standardized paths were significant (*p* < 0.05), except for the path from behavioral attitudes to behavioral intention, which did not reach significance. Perceived autonomy support positively predicted BPN (*β* = 0.761, *p* < 0.001), and exercise persistence (*β* = 0.435, *p* < 0.001), supporting H1. BPN positively predicted behavioral attitudes (*β* = 0.589, *p* < 0.001), subjective norms (*β* = 0.623, *p* < 0.001), perceived behavioral control (*β* = 0.741, *p* < 0.001) and exercise persistence (*β* = 0.396, *p* < 0.001), consistent with H2. Subjective norms (*β* = 0.110, *p* < 0.01) and perceived behavioral control (*β* = 0.510, *p* < 0.001) significantly predicted behavioral intentions, whereas behavioral attitudes did not, yielding partial support for H3 and partial rejection of H5 and H6. Moreover, both perceived behavioral control (*β* = 0.092, *p* < 0.01) and behavioral intention (*β* = 0.062, *p* < 0.05) positively predicted exercise persistence, supporting H4.

Mediation analyses (see Supplementary Table S1) further demonstrated that perceived autonomy support indirectly influenced behavioral attitudes, subjective norms, and perceived behavioral control through BPN, with effect sizes of 0.448, 0.474, and 0.564, respectively; BPN indirectly affected behavioral intention via subjective norms and perceived behavioral control, with effect sizes of 0.069 and 0.378, respectively. BPN also indirectly predicted exercise persistence through perceived behavioral control (*β* = 0.068, *p* < 0.01). In additional, subjective norms and perceived behavioral control each mediated the effect of behavioral intention on exercise persistence, with effect sizes of 0.007 and 0.032, respectively. Perceived autonomy support indirectly predicted exercise persistence through BPN alone and through the sequential pathway involving BPN and TPB constructs, with effect sizes of 0.302 and 0.074, respectively. All 95% CIs did not contain 0, confirming significant mediation effects and supporting parts of H5–H7 and H8–H10.

**Figure 3:**
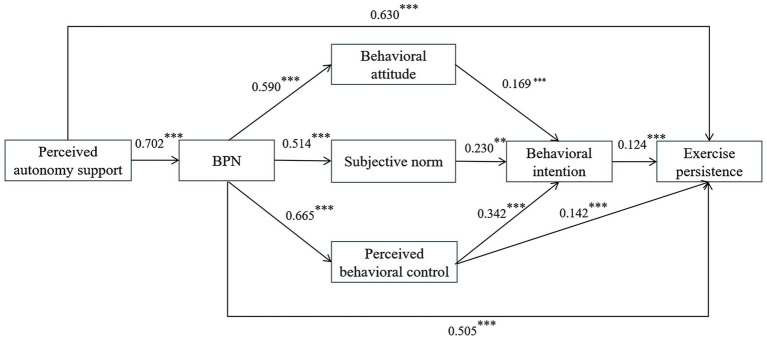
Standardized path coefficients of the SEM of hypothesized relationships between variables in the model for senior high school students.

As depicted in Figure 3, all direct paths for the overall sample of senior high school students reached statistical significance (*p* < 0.01). Specifically, perceived autonomy support positively predicted BPN (*β* = 0.702, *p* < 0.001) and exercise persistence (*β* = 0.630, *p* < 0.001), supporting H1. In turn, BPN positively predicted behavioral attitudes (*β* = 0.590, *p* < 0.001), subjective norms (*β* = 0.514, *p* < 0.001), perceived behavioral control (*β* = 0.665, *p* < 0.001), and exercise persistence (*β* = 0.505, *p* < 0.001), consistent with H2. Behavioral attitudes (β = 0.169, *p* < 0.001), subjective norms (β = 0.230, *p* < 0.001), and perceived behavioral control (*β* = 0.342, *p* < 0.001) all significantly predicted behavioral intention, supporting H3. Moreover, both perceived behavioral control (*β* = 0.142, *p* < 0.001) and behavioral intention (*β* = 0.124, *p* < 0.001) directly predicted exercise persistence, supporting H4.

Mediation analyses (see Supplementary Table S2) further revealed that perceived autonomy support indirectly influenced behavioral attitudes, subjective norms, and perceived behavioral control through BPN, with effect sizes of 0.414, 0.361, and 0.467, respectively. BPN indirectly affected behavioral intention via behavioral attitude (*β* = 0.100, *p* < 0.01), subjective norms (*β* = 0.118, *p* < 0.001), and perceived behavioral control (*β* = 0.227, *p* < 0.01). BPN also exerted an indirect effect on exercise persistence through perceived behavioral control (*β* = 0.095, *p* < 0.001). Furthermore, behavioral attitudes, subjective norms, and perceived behavioral control each indirectly predicted exercise persistence through behavioral intention, with effect sizes of 0.021, 0.028, and 0.042, respectively. Perceived autonomy support additionally predicted exercise persistence through BPN alone (*β* = 0.355, *p* < 0.001) and through the sequential mediation of BPN and TPB constructs (*β* = 0.105, *p* < 0.001). For all mediation pathways, the 95% confidence intervals excluded zero, indicating statistically significant indirect effects and supporting H5-H10.

In summary, the integrated BPN-TPB model revealed stage-specific differences in how perceived autonomy support shape exercise persistence. Notably, behavioral attitudes significantly predicted behavioral intention among senior high school students but not among junior high school students; thus, supporting H11.

#### Mediation analysis for male and female senior high school students

**Figure 4:**
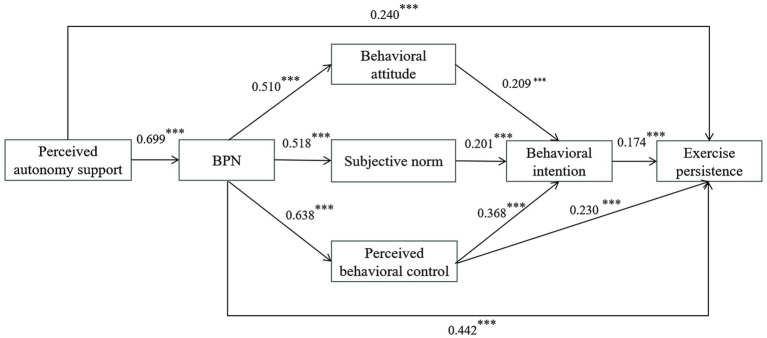
Standardized path coefficients of the SEM of hypothesized relationships between factors in the model for male senior high school students.

The above findings suggested that gender may moderate the effect of perceived autonomy support on exercise persistence among senior high school students, thereby necessitating separate mediation analyses for males and females. As illustrated in Figure 4, all standardized path coefficients in the male model were statistically significant (*p* < 0.01). Perceived autonomy support positively predicted BPN (*β* = 0.699, *p* < 0.001) and exercise persistence (*β* = 0.240, *p* < 0.001), supporting H1. In turn, BPN positively predicted behavioral attitudes (*β* = 0.510, *p* < 0.001), subjective norms (*β* = 0.518, *p* < 0.001), perceived behavioral control (*β* = 0.638, *p* < 0.001), and exercise persistence (*β* = 0.442, *p* < 0.001), supporting H2. Behavioral attitudes (*β* = 0.209, *p* < 0.001), subjective norms (*β* = 0.201, *p* < 0.001), and perceived behavioral control (β = 0.368, *p* < 0.001) each positively predicted behavioral intention, consistent with H3. Moreover, both perceived behavioral control (*β* = 0.230, *p* < 0.001) and behavioral intention (*β* = 0.174, *p* < 0.001) positively predicted exercise persistence, confirming H4.

Mediation analyses (see Supplementary Table S3) further revealed that perceived autonomy support indirectly influenced behavioral attitudes, subjective norms, and perceived behavioral control through BPN, with effect sizes of 0.357, 0.362, and 0.446, respectively; BPN indirectly predicted behavioral intention via behavioral attitudes (*β* = 0.107, *p* < 0.01), subjective norms (*β* = 0.104, *p* < 0.001), and perceived behavioral control (*β* = 0.235, *p* < 0.001). BPN also exerted an indirect effect on exercise persistence through perceived behavioral control (*β* = 0.147, *p* < 0.001). Additionally, behavioral attitudes, subjective norms, and perceived behavioral control each indirectly influenced exercise persistence via behavioral intention, with effect sizes of 0.036, 0.035, and 0.064, respectively. Finally, perceived autonomy support indirectly predicted exercise persistence through BPN alone (*β* = 0.309, *p* < 0.001) and through the sequential mediation of BPN and TPB constructs (*β* = 0.157, *p* < 0.001). All 95% CIs excluded zero, indicating significant mediation effects and supporting H5-H10.

**Figure 5:**
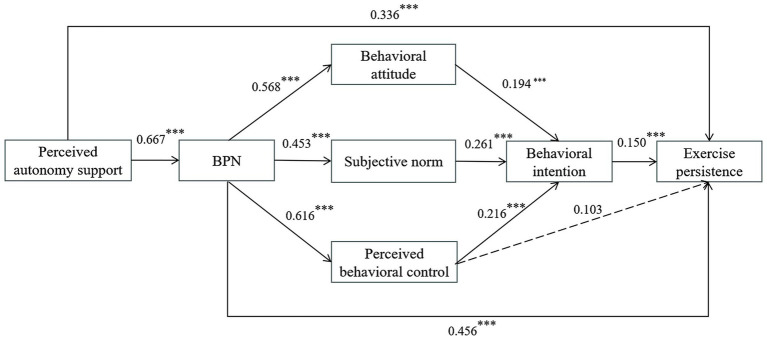
Standardized path coefficients of the SEM of hypothesized relationships between factors in the model for female senior high school students.

The mediation analysis for female students (Figure 5) revealed that nearly all direct paths were significant (*p* < 0.01), except for the path from perceived behavioral control to exercise persistence. Perceived autonomy support positively predicted BPN (*β* = 0.667, *p* < 0.001) and exercise persistence (*β* = 0.336, *p* < 0.001), supporting H1. In turn, BPN positively predicted behavioral attitudes (*β* = 0.568, *p* < 0.001), subjective norms (*β* = 0.453, *p* < 0.001), perceived behavioral control (*β* = 0.616, *p* < 0.001), and exercise persistence (*β* = 0.456, *p* < 0.001), consistent with H2. Behavioral attitudes (*β* = 0.194, *p* < 0.01), subjective norms (β = 0.261, *p* < 0.001), and perceived behavioral control (*β* = 0.216, *p* < 0.01) each significantly predicted behavioral intention, supporting H3. Behavioral intention (*β* = 0.150, *p* < 0.001) positively predicted exercise persistence, whereas perceived behavioral control did not, offering only partial support for H4 and disconfirming H8.

Mediation analyses (see Supplementary Table S4) further showed that perceived autonomy support indirectly affected behavioral attitudes (*β* = 0.379, *p* < 0.001), subjective norms (*β* = 0.302, *p* < 0.001), and perceived behavioral control (*β* = 0.411, *p* < 0.001) through BPN. Behavioral attitudes, subjective norms, and perceived behavioral control each indirectly influenced exercise persistence via behavioral intention, with effect sizes of 0.029, 0.039, and 0.032, respectively. Perceived autonomy support also indirectly predicted exercise persistence through BPN alone (*β* = 0.305, *p* < 0.001) and through the sequential mediation of BPN and TPB constructs (*β* = 0.079, *p* < 0.001). All 95% CIs excluded zero, confirming significant mediation effects and supporting H5–H10.

In summary, the integrated BPN-TPB pathway from perceived autonomy support to exercise persistence exhibited gender-specific patterns. Among male students, perceived behavioral control exerted a direct influence on exercise persistence, whereas among female students it contributed only indirectly through behavioral intention, showing no direct effect on exercise persistence.

## Discussion

The findings demonstrate that perceived autonomy support is a significant predictor of exercise persistence, consistent with prior research indicating that adolescents are more likely to form positive and stable exercise habits when families, schools, and communities foster a supportive climate and provide diverse facilities and resources (Chen and Zhu, 2022; Hong and Li, 2022). Students who perceive autonomy support from physical education teachers also exhibit greater effort and persistence toward their exercise goals, stronger willingness to tackle challenging, and enhanced exercise experiences and self-efficacy, all of which contribute to higher levels of exercise commitment (Rekaa et al., 2019). Our results not only confirm autonomy support as a key determinant of adolescents’ exercise persistence but also indicate a moderate effect size, aligning with findings reported by Fang et al. (2020). Although amount of explained variance in our study (57.8% for junior high school students and 47.6% for senior high school students, controlling for gender, age, and grade) differs from the 30.1% reported by Fang et al. (2020) in a sample of sophomore college students (controlling only for gender), both sets of results reached statistical significance. These discrepancies likely stem from differences in sample characteristics and covariate inclusion. Nonetheless, both studies highlight the critical role of autonomy-supportive, self-directed exercise environments in sustaining students’ exercise persistence.

The results indicate that BPN partially mediates the relationship between perceived autonomy support and exercise persistence. Prior evidence suggests that encouragement from teachers, parents, and peers helps students recognize the value of physical activity for healthy development, satisfies their need for relatedness, enhances self-confidence, and facilitates full engagement in exercise (Zhou et al., 2019). In recent years, Chinese secondary schools have increasingly emphasized autonomy in physical education by offering diverse course options, establishing sports club, and improving facilities (Fang et al., 2020). These initiatives provide adolescents with greater freedom to choose when, how, and what to exercise, thereby enhancing their intrinsic motivation. Consistent with previous work (Kang et al., 2020; Zhang and Dong, 2017), higher BPN satisfaction is associated with greater persistence in physical activity. By enhancing intrinsic motivation and fostering stronger exercise intentions, BPN satisfaction encourages sustained participation and persistence (Adelusi et al., 2023). Adolescents who experience higher levels of BPN satisfaction exhibit greater autonomy and initiative, which facilitates long-term adhere to exercise behaviors (Rodrigues et al., 2020), a relationship further supported by increased self-efficacy and self-drive motivation (Bentzen and Malmquist, 2022). According to SDT, BPN are innate human motivators that promote individuals to seek contexts that fulfill these needs (Deci and Ryan, 2000; Ryan, 1995). Thus, an autonomy-supportive environment enhances BPN satisfaction, which in turn promotes the initiation and maintenance of persistent exercise behavior.

The results indicate that BPN not only exert positive effects on the TPB antecedents but also indirectly influence behavioral intention and exercise persistence through these constructs, aligning with prior research and with theoretical models integrating BPN into TPB (Hagger et al., 2006). Whereas TPB outlines the social-cognitive determinants of exercise persistence, BPN reflects individuals’ affective evaluations of their social environment. For example, Gucciardi and Jackson (2015) using Bayesian SME, demonstrated that fulfilling BPN enhances both enjoyment of and persistence in physical activity. From a hierarchical motivation perspective, these findings underscore the top-down influence exerted by situational perceptions of autonomy support and relatedness on TPB constructs. Consequently, behavioral intentions energized by BPN translate into more committed, goal-directed, and sustained exercise behaviors.

BPN exert significant effects on the antecedents of the TPB, with the strongest influence on perceived behavioral control, followed by behavioral attitudes, and a comparatively weaker effect on subjective norms. This pattern aligns with findings reported by Jang et al. (2021). Individuals with higher levels of BPN satisfaction are more likely to internalize external perspectives and place greater value on others’ opinions when making decisions (Harris and Hagger, 2007). In addition, Cho et al. (2023) found that need satisfaction enhances individuals’ positive attitudes toward target behaviors, thereby promoting more adaptive behavioral outcomes. When psychological needs are met, individuals engage in physical activity more consciously and systematically, demonstrating stronger self-regulation. In contrast, subjective norms primarily reflect the perceived expectations of significant others regarding exercise. Within the TPB framework, behavioral attitudes, subjective norms, and perceived behavioral control each directly predict behavioral intention (Yin et al., 2018). These findings suggest that fostering more positive health-related attitudes, strengthening adolescents’ identification with health behaviors, and enhancing their perceived behavioral control may be particularly effective for shaping their intentions to participate in physical activity.

The present study also found that perceived behavioral control and behavioral intention significantly influence exercise behavior, consistent with findings by Husebø et al. (2013). Specifically, higher perceived behavioral control strengthens adolescents’ exercise intentions, which in turn increases their likelihood of engaging in physical activity. Prior research indicates that a strong behavioral intention fosters positive emotional experiences, including self-satisfaction, confidence, and a sense of accomplishment, which further bolster exercise persistence (Rodrigues et al., 2019). Similarly, de Bruijn et al. (2014) showed individuals with high perceived behavioral control tend to form stronger exercise intentions, aligning with the TPB assertion that both actual behavioral control and behavioral intention directly shape behavior (Kashif et al., 2018). From an applied perspective, these findings highlight the importance of satisfying basic psychological needs in physical education. For example, teachers should evaluate students’ learning contexts and individual needs, design large-unit, student-centered curricula, and implement diverse, ongoing assessments to monitor engagement and adjust instruction in real time. Overall, our results support the integrated BPN–TPB model, indicating that perceived autonomy support influences exercise persistence both directly and indirectly through BPN satisfaction and TPB pathways.

Interestingly, our findings reveal that the mechanism underlying exercise persistence vary across educational stages. Among senior high school students, behavioral attitudes exerted a significant direct effect on behavioral intention, whereas this pathway was not apparent in junior high school students. This pattern aligns with Nie and Dong (2015) but contrasts with findings reported by Viksi and Tilga (2022). Behavioral attitudes refer to evaluative beliefs regarding the positive or negative outcomes of a particular behavior (Ajzen, 2002), and individuals are more likely to engage in health behaviors when they believe that these behaviors will produce desired outcome (Bandura, 2004). Senior students, having undergone substantial cognitive development, identity formation, and changes in social relationships, are better able to independently and confidently assess their behavioral attitudes, which in turn shapes their exercise intentions. In contrast, junior students are in a stage of growth and exploration, during which their behavioral attitudes are more strongly influenced by external factors, such as peer pressure, family environment, and situational motivation. This reliance on external influences may contribute to a more complex and less stable relationship between behavioral attitudes and behavioral intentions (Xie et al., 2009).

The results also revealed a significant gender effect on exercise persistence among senior high school students. Even after controlling for other factors, gender remained significant, suggesting that the mechanisms underlying exercise persistence differ between male and female students. SEM analysis further indicated that perceived behavioral control directly predicted exercise persistence in male students, but not in female students. Possible reasons include: (1) gender differences, as social, cultural, and family expectations may shape females’ cognition and evaluation of behavioral control (Chen et al., 2017). (2) psychological mechanisms, with factors such as self-efficacy, motivation, and health awareness exerting strong influence in females, potentially diminishing the direct effect of perceived behavioral control and thereby reducing its direct effect; and (3) social environmental influence, including variations in social support, family context, and peer influence, which may differentially affect exercise persistence across genders. These findings highlight the need to consider gender-specific mechanisms when designing interventions to promote exercise persistence in adolescents.

Additionally, this study has several limitations. First, its cross-sectional design precludes causal inference between perceived autonomy support and exercise persistence; longitudinal or experimental studies are needed to clarify the direction and strength of these relationships over time. Second, although a simplified stratified random sampling method was used, the sample may not fully represent adolescents from diverse socioeconomic backgrounds or geographic regions (e.g., urban versus rural), which limits the generalizability of our findings. Finally, our measure of “perceived autonomy support” combined all sources (parents, teachers, peers) without distinguishing among them; future research should explore how autonomy support from specific providers differentially affects adolescents’ exercise persistence.

## Conclusions and recommendations

### Conclusion

BPN are relevant across diverse cultures and life domains, yet their application in sports contexts remain unexplored. This study contributes to existing literature by examining self-efficacy, BPN, TPB, and exercise persistence as an integrated system. Our findings indicate that perceived autonomy support is directly and positively associated with adolescents’ exercise persistence. Both BPN alone and the sequential pathway from BPN to TPB constructs as mediators in this relationship. Notably, behavioral attitudes significantly predict behavioral intention among senior high school students but do not exert a significant effect among junior high school students. Additionally, perceived behavioral control significantly predicts exercise persistence among male senior high school students, while this association is not significant in females.

### Recommendations

Future research should clarify the psychological and social mechanisms underlying exercise persistence across different educational stages and genders and develop targeted interventions to bolster adolescents’ long-term engagement in physical activity. Physical education teachers should align curricular objectives with students’ exercise needs and academic goals, intentionally structure learning experiences, and implement diverse, innovative strategies to satisfy BPN and strengthen exercise intentions. At a systemic level, schools and government agencies should establish a “family–school–community” collaborative framework by expanding extracurricular activity spaces, integrating on-campus and local resources, and strengthening cooperation among families, schools, and community organizations. Within this framework, schools lead, families actively participate, and communities provide supportive collaboration, collectively fostering adolescents’ autonomy, perceived support, and ultimately promoting sustained physical-activity habits and exercise persistence nationwide.

## Data Availability

The datasets presented in this study can be found in online repositories. The names of the repository/repositories and accession number(s) can be found in the article/Supplementary material.
