# Supplementary material for: Effect of perceived autonomy supports on exercise persistence for adolescents: an integrated model based on basic psychological needs theory and the theory of planned behavior
Source: Front Psychol. 2025 Dec 11;16:1692940. doi: 10.3389/fpsyg.2025.1692940 (PMC12739754; doi:10.3389/fpsyg.2025.1692940)
Supplement: Supplementary file 1 [file Table_1.docx]

Supplementary Table S1. Results of mediation effects among variables in the model for junior students

| Path | Effect size | 95% CI | | P |
| --- | --- | --- | --- | --- |
|  |  | Upper limit | Lower limit |  |
| Perceived autonomy support→BPN→Behavioral attitude | 0.448 | 0.411 | 0.485 | .000 |
| Perceived autonomy support→BPN→Subjective norms | 0.474 | 0.442 | 0.505 | .000 |
| Perceived autonomy support→BPN→Perceived behavioral control | 0.564 | 0.521 | 0.603 | .000 |
| BPN→Subjective norms→Behavioral intention | 0.069 | 0.035 | 0.103 | .000 |
| BPN→Perceived behavioral control→Behavioral intention | 0.378 | 0.306 | 0.45 | .000 |
| BPN→Perceived behavioral control→Exercise persistence | 0.068 | 0.021 | 0.114 | .003 |
| Subjective norms→Behavioral intention→Exercise persistence | 0.007 | 0.002 | 0.014 | .001 |
| Perceived behavioral control→Behavioral intention→Exercise persistence | 0.032 | 0.013 | 0.051 | .001 |
| Perceived autonomy support→BPN→Exercise persistence | 0.302 | 0.243 | 0.36 | .000 |
| Perceived autonomy support→BPN→TPB→Exercise persistence | 0.074 | 0.043 | 0.105 | .000 |
