# Supplementary material for: Effect of perceived autonomy supports on exercise persistence for adolescents: an integrated model based on basic psychological needs theory and the theory of planned behavior
Source: Front Psychol. 2025 Dec 11;16:1692940. doi: 10.3389/fpsyg.2025.1692940 (PMC12739754; doi:10.3389/fpsyg.2025.1692940)
Supplement: Supplementary file 2 [file Table_2.docx]

Supplementary Table S2. Results of mediation effects among variables in the model for senior students

| Path | Effect size | 95% CI | | P |
| --- | --- | --- | --- | --- |
|  |  | Upper limit | Lower limit |  |
| Perceived autonomy support→BPN→Behavioral attitude | 0.414 | 0.372 | 0.456 | 0.000 |
| Perceived autonomy support→BPN→Subjective norms | 0.361 | 0.320 | 0.403 | 0.000 |
| Perceived autonomy support→BPN→Perceived behavioral control | 0.467 | 0.420 | 0.511 | 0.000 |
| BPN→Behavioral attitude→Behavioral intention | 0.100 | 0.041 | 0.157 | 0.001 |
| BPN→Subjective norms→Behavioral intention | 0.118 | 0.089 | 0.152 | 0.000 |
| BPN→Perceived behavioral control→Behavioral intention | 0.227 | 0.156 | 0.304 | 0.001 |
| BPN→Perceived behavioral control→Exercise persistence | 0.095 | 0.054 | 0.138 | 0.000 |
| Behavioral attitude→Behavioral intention→Exercise persistence | 0.021 | 0.008 | 0.038 | 0.001 |
| Subjective norms→Behavioral intention→Exercise persistence | 0.028 | 0.018 | 0.041 | 0.000 |
| Perceived behavioral control→Behavioral intention→Exercise persistence | 0.042 | 0.027 | 0.062 | 0.000 |
| Perceived autonomy support→BPN→Exercise persistence | 0.355 | 0.126 | 0.394 | 0.000 |
| Perceived autonomy support→BPN→TPB→Exercise persistence | 0.105 | 0.043 | 0.162 | 0.000 |
