# Supplementary material for: Effect of perceived autonomy supports on exercise persistence for adolescents: an integrated model based on basic psychological needs theory and the theory of planned behavior
Source: Front Psychol. 2025 Dec 11;16:1692940. doi: 10.3389/fpsyg.2025.1692940 (PMC12739754; doi:10.3389/fpsyg.2025.1692940)
Supplement: Supplementary file 3 [file Table_3.docx]

Supplementary Table S3. Results of mediation effects among variables in the model for senior male students

| Path | Effect size | 95% CI | | P |
| --- | --- | --- | --- | --- |
|  |  | Upper limit | Lower limit |  |
| Perceived autonomy support→BPN→Behavioral attitude | 0.357 | 0.290 | 0.419 | 0.000 |
| Perceived autonomy support→BPN→Subjective norms | 0.362 | 0.302 | 0.424 | 0.000 |
| Perceived autonomy support→BPN→Perceived behavioral control | 0.446 | 0.375 | 0.513 | 0.000 |
| BPN→Behavioral attitude→Behavioral intention | 0.107 | 0.034 | 0.183 | 0.005 |
| BPN→Subjective norms→Behavioral intention | 0.104 | 0.058 | 0.153 | 0.000 |
| BPN→Perceived behavioral control→Behavioral intention | 0.235 | 0.140 | 0.352 | 0.000 |
| BPN→Perceived behavioral control→Exercise persistence | 0.147 | 0.095 | 0.21 | 0.000 |
| Behavioral attitude→Behavioral intention→Exercise persistence | 0.036 | 0.010 | 0.073 | 0.005 |
| Subjective norms→Behavioral intention→Exercise persistence | 0.035 | 0.018 | 0.057 | 0.000 |
| Perceived behavioral control→Behavioral intention→Exercise persistence | 0.064 | 0.038 | 0.099 | 0.000 |
| Perceived autonomy support→BPN→Exercise persistence | 0.309 | 0.213 | 0.346 | 0.000 |
| Perceived autonomy support→BPN→TPB→Exercise persistence | 0.157 | 0.072 | 0.189 | 0.000 |
