# Supplementary material for: Effect of perceived autonomy supports on exercise persistence for adolescents: an integrated model based on basic psychological needs theory and the theory of planned behavior
Source: Front Psychol. 2025 Dec 11;16:1692940. doi: 10.3389/fpsyg.2025.1692940 (PMC12739754; doi:10.3389/fpsyg.2025.1692940)
Supplement: Supplementary file 4 [file Table_4.docx]

Supplementary Table S4. Results of mediation effects among variables in the model for senior female students

| Path | Effect size | 95% CI | | P |
| --- | --- | --- | --- | --- |
|  |  | Upper limit | Lower limit |  |
| Perceived autonomy support→BPN→Behavioral attitude | 0.379 | 0.32 | 0.439 | 0.000 |
| Perceived autonomy support→BPN→Subjective norms | 0.302 | 0.245 | 0.362 | 0.000 |
| Perceived autonomy support→BPN→Perceived behavioral control | 0.411 | 0.343 | 0.478 | 0.000 |
| BPN→Behavioral attitude→Behavioral intention | 0.110 | 0.044 | 0.176 | 0.002 |
| BPN→Subjective norms→Behavioral intention | 0.118 | 0.081 | 0.164 | 0.000 |
| BPN→Perceived behavioral control→Behavioral intention | 0.133 | 0.057 | 0.230 | 0.001 |
| Behavioral attitude→Behavioral intention→Exercise persistence | 0.029 | 0.009 | 0.057 | 0.001 |
| Subjective norms→Behavioral intention→Exercise persistence | 0.039 | 0.022 | 0.062 | 0.000 |
| Perceived behavioral control→Behavioral intention→Exercise persistence | 0.032 | 0.016 | 0.059 | 0.000 |
| Perceived autonomy support→BPN→Exercise persistence | 0.305 | 0.222 | 0.402 | 0.000 |
| Perceived autonomy support→BPN→TPB→Exercise persistence | 0.079 | 0.031 | 0.092 | 0.000 |
